# Prenatal gunshot wound, a rare cause of maternal and fetus trauma, a case report

**DOI:** 10.1016/j.ijscr.2019.05.034

**Published:** 2019-05-28

**Authors:** Gabriel A. Molina, William G. Aguayo, J. Marcelo Cevallos, Patricio F. Gálvez, Juan F. Calispa, Kevin A. Arroyo, Lenin J. Guzmán, María M. Cobo, Bernardo M. Gutierrez, Ronald T. Toapanta, María M. Briceño

**Affiliations:** a Pontificia Universidad Católica del Ecuador PUCE, Quito, Ecuador; b Department of General Surgery, Hospital San Francisco, IESS, Quito, Ecuador; c Hospital de Especialidades Fuerzas Armadas, Quito, Ecuador; d Universidad Central del Ecuador, Quito, Ecuador; e Universidad San Francisco de Quito, College of Biological and Environmental Sciences, USFQ, Quito, Ecuador

**Keywords:** Maternal trauma, Gunshot wound, Fetal trauma, Case report

## Abstract

**Background:**

Gunshot wounds in pregnant women, although rare, represent an important cause of fetal and maternal mortality. Understanding the mechanism of injury is essential to identify the possible injuries and to adequately manage the complexity of these emergency scenarios.

**Case presentation:**

We present a case of a 27-year-old woman and her 37 week fetus who were the victims of a gunshot wound. The trajectory of the bullet injured not only the mother but also the developing fetus. An emergency c-section was performed and the bullet was removed from the infant's abdomen. The two patients fully recovered and on follow up controls both patients are doing well.

**Conclusions:**

Trauma events, and particularly gunshot wounds in pregnant women requires promptly and adequate intervention. Coordinated efforts from multidisciplinary clinical teams are needed as well as the appropriate training in maternal and infant resuscitative measures and surgical techniques.

## Highlights

• Trauma during pregnancy is an important cause of adverse fetal and maternal outcomes.
• Gunshot wounds to the gravid uterus are generally lethal for the fetus, and cause significant morbidity to the mother.
• Gunshot wounds in a pregnant woman must be handled by a multidisciplinary team.
• Training in maternal and infant resuscitative measures and surgical techniques are vital.

## 1 Introduction

Trauma affects 1 in 12 pregnant women and has a notable impact on maternal mortality, morbidity and the outcome of the pregnancy, with fetal loss occurring in 0.03–0.09% of these cases [1]. Blunt trauma is the most common mechanism of trauma (69%) among the pregnant population, with car accidents at the top of the list. Other common causes of blunt trauma are falls (22%), assaults (22%), and burns (1%) [2]. Penetrating trauma accounts for less than 2% of all pregnant trauma admittances. Of these, 73% were gunshot, 23% stab wounds and 4% shotgun-related. Gunshots wounds are associated with a maternal visceral injury between 19–38%, [3]. Unfortunately, the fetus has a worse prognosis, with an injury rate of up to 80% and a perinatal mortality rate between 41–71% [4].

The impact of maternal abdominal trauma on the fetus is highly associated with the gestational age at the time of the trauma. During the first 12 weeks of gestation, direct injury to the fetus is less likely due to the protective effect of the bony pelvis [5]. However, the risk for the fetus after a gunshot to the mother’s abdomen increases with the progression of the pregnancy, as maternal anatomical and physiological changes accompany the fetus development. In all cases, trauma injuries during pregnancy affect both the fetus and the mother, and their management and clinical care represent a challenge for any medical team. The prevalence of interpersonal violence is affected by several aspects, including socioeconomic factors, cultural upbringing, the status of women in the specific society and the normative use of violence in conflict situation. Regrettably, violence due to the use of firearms has escalated considerably in Latin America in recent years, exposing more women and pregnant women to trauma injuries as a result of violent crimes.

This the work has been reported in line with the SCARE criteria [11].

## 2 Case presentation

**Fig. 1:**
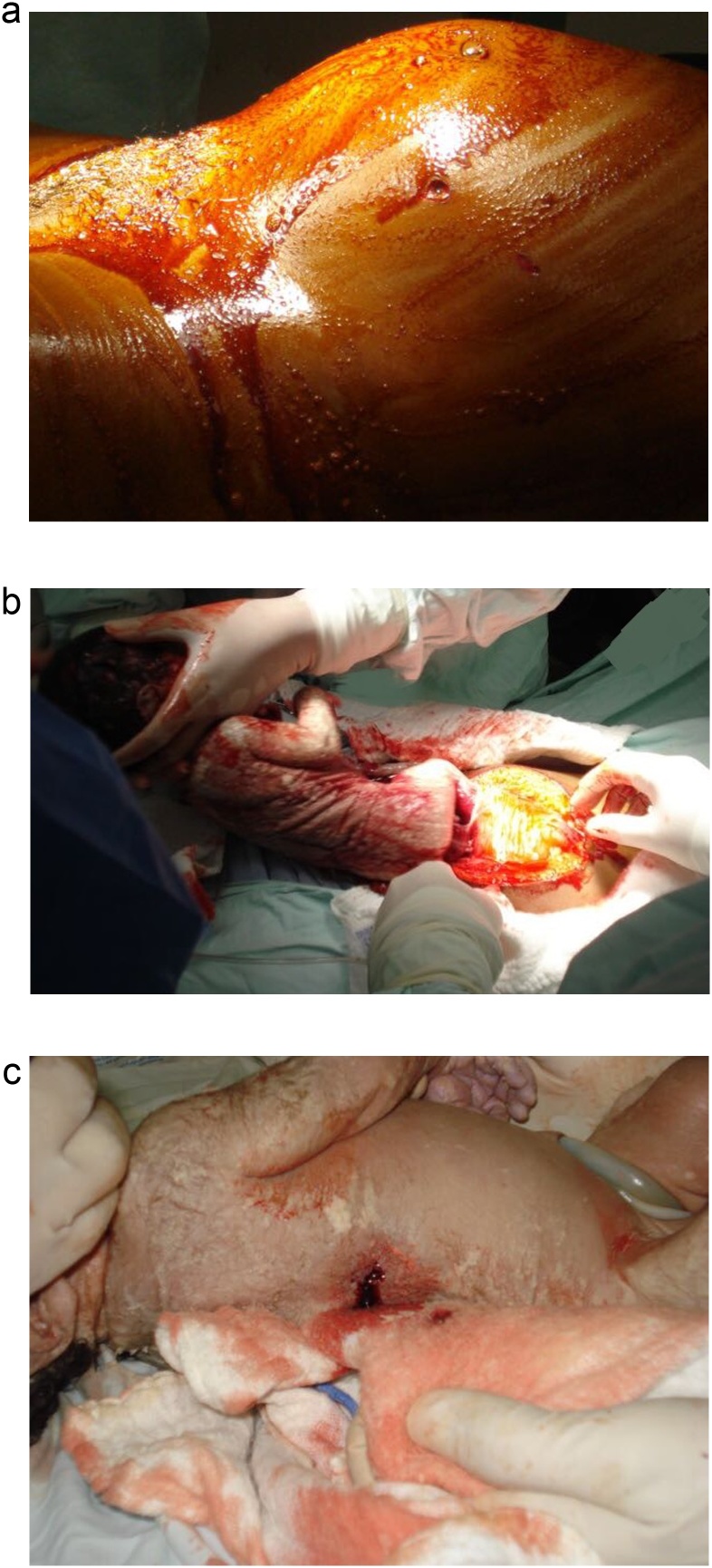
(A): Bullet entrance wound, in the lower left abdomen. (B): Emergency cesarean section. (C): Penetrating entry wound in the infant’s right thoracoabdominal region.

A 27-year-old woman without past medical history in her 37th week of pregnancy was the victim of a gunshot wound to her lower abdomen while being robbed and attacked by two unidentified burglars. The patient was brought by paramedic personnel to the emergency room 30 min after the violent attack. Upon arrival, a tachycardic and hypotensive patient was encountered. On examination, she presented a 50/30 blood pressure without palpable peripheral pulses; nonetheless, the fetus was noted to be active. The women’s abdomen was diffusely tender and rigid, and a single bullet entrance wound, without an exit wound in the lower left abdomen was seen (Fig. 1A). The cervix was closed, and no blood was found on rectal examination. Patient was reanimated and transported immediately to the operating room for an emergency laparotomy by a team of general surgeons, obstetricians, pediatricians, and pediatric surgeons.

Under general anesthesia at laparotomy, a 1 × 0.5 cm gunshot injury to the uterine fundus along with 300cc of clear amniotic fluid with whitish lumps and 700cc of blood clots were discovered in her abdomen. After a thorough exploration, no other injury was identified, and no bullet or fragment was found. An extensive peritoneal lavage was completed and an emergency cesarean section was performed (Fig. 1B).

**Fig. 2:**
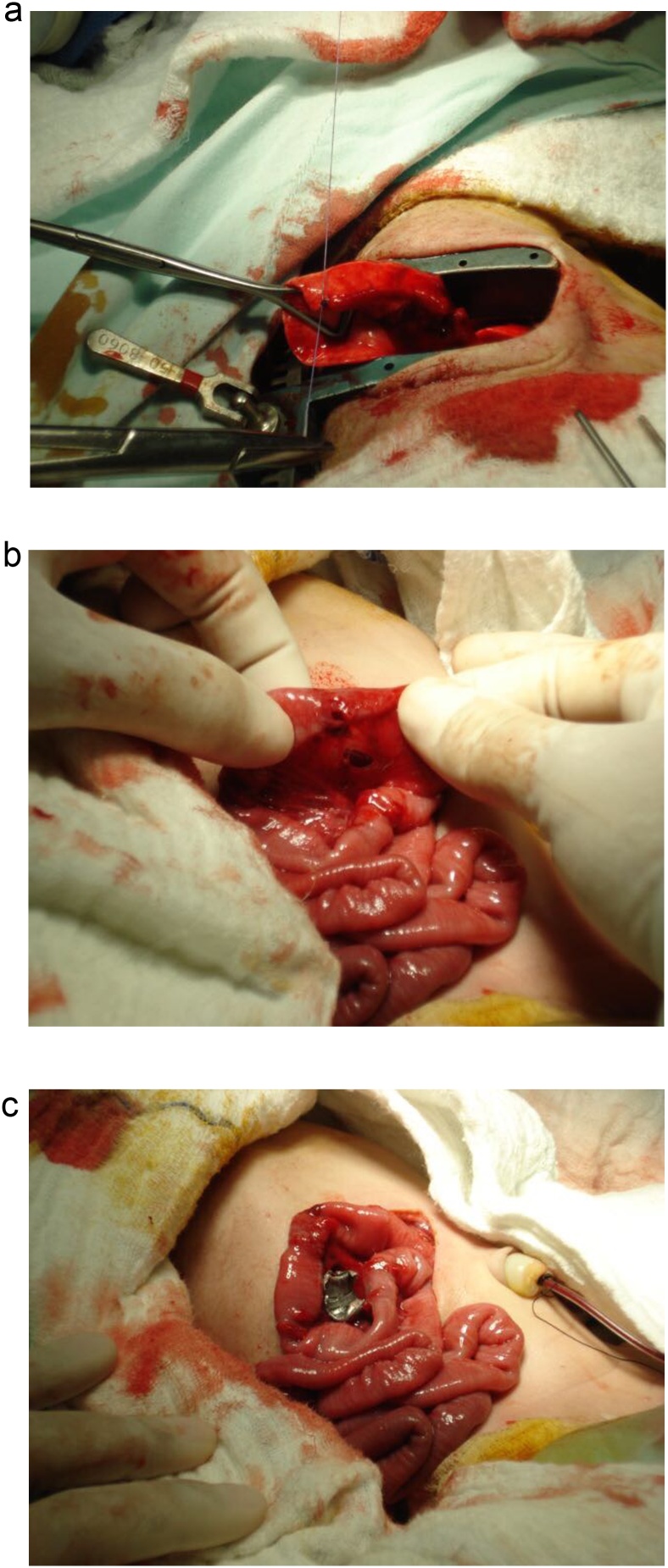
A: Lung laceration to the infant's right lung. (B): Bowel lesion found in the infant's abdomen (C): Bullet found near the root of the infant's mesentery between the intestinal loops.

A 2600 g male infant with a 6 Apgar score was delivered. During reanimation, the infant presented with severe respiratory distress and a penetrating entry wound in the infant’s right thoracoabdominal region without an exit wound was seen (Fig. 1C). Due to the nature of the injuries, an emergency consultation with the pediatric surgeon was required. A right posterolateral thoracotomy was performed. A 5 × 5 mm laceration to the inferior lobe of the right lung and a 1 × 0.5 cm right diaphragmatic injury were discovered (Fig. 2A). However, the bullet was nowhere to be found in the thorax. A diaphragmatic lesion was noticed, and the abdominal cavity was explored. Intestinal fluid (50 cc) was found in the infant’s abdomen along with a 2 cm bowel injury, 50 cm distal to the ligament of Treitz that encompassed 30% of the intestinal circumference. (Fig. 2B). The rest of the abdomen appeared normal, and the 2 × 1 cm bullet was found near the root of the mesentery between the intestinal loops (Fig. 2C).

**Fig. 3:**
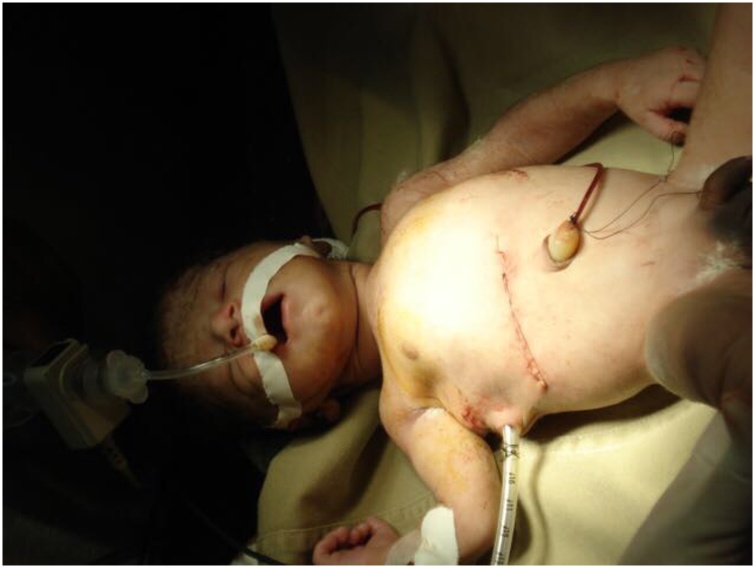
Thoracic drainage and surgical wound in the infant.

With these findings, surgery was straightforward. Primary closure of the infants’ pulmonary and diaphragmatic injury was completed, and a right chest tube was set up. The extraction of the bullet was accomplished without difficulties. After that, a thorough lavage of the baby’s peritoneal cavity was performed, followed by debridement of the injured bowel and a primary closure in a single layer fashion with an absorbable suture (Vicryl, Ethicon, Johnson & Johnson, Seoul, Korea) (Fig. 3). Following surgery, the mother was stable and transferred to the ward for postoperative care and recovery while the neonate was admitted to the intensive care unit (ICU) for further postoperative care and respiratory support.

Breast milk diet was initiated on the second postoperative day, and as the infant showed a good clinical development, thoracic drainage was removed on the 6th postoperative day, and the infant was transferred from the ICU to the general ward with his mother. After a 2-week cycle of broad-spectrum antibiotics, our patient and her newborn were discharged in good health and no further treatment was prescribed. In follow up controls, after 5 months, the two patients were doing well.

## 3 Discussion

Trauma during pregnancy is the leading cause of non-obstetric maternal mortality. It causes severe adverse fetal and maternal events, with up to 20% of maternal deaths directly attributable to trauma injuries [1]. Trauma injuries warrant a special consideration when a pregnant woman is affected, due to maternal physiology and anatomy particularities. During pregnancy, there is an increase in blood volume by 50%, an increase in 15 beats per minute in the normal heart rate and a state of hypotension because of the vasodilator effect of progesterone [2,3]. Moreover, the abdominal viscera are displaced to allow for the progressive size increase of the uterus [5].

Fetal loss after trauma often develops from prolonged maternal hypotension, placental abruption, uterine rupture, direct trauma to the uterus, or maternal death [6]. Penetrating trauma cases during pregnancy are rare, and account only for 1.5% of episodes of trauma during pregnancy. However, abdominal injuries, if occurring during the third trimester of pregnancy, are associated with high maternal morbidity (60–70%) and a particularly high fetal death rate (71%) [5,7].

This case presents a rare gunshot injury with a positive outcome, a fully recovered mother and her baby surviving a prenatal penetrating gunshot trauma. There are only a few other cases similar to this one in the literature having in common a woman in their third trimester of pregnancy [8,9].

It is likely that after the gunshot, the uterine musculature, amniotic fluid, and fetus absorbed a great amount of energy from the bullet [3], providing protection to the mother and reducing the potential damage to other maternal organs [1,4]. Visceral injury in penetrating abdominal trauma is only 15% to 40% compared with 80% to 90% in non-pregnant women. Yet the results on the fetus are often fatal [1].

The particular trajectory of the bullet and position of the fetus at the moment of the injury benefitted the final outcome, as well as the fast and effective response from the medical team.

When an injury happens to a pregnant woman, there are two potential victims at the same time. That is why in 1996, Morris et al, defined the criteria for “salvageable infant”; referring to equal or greater gestational age than 26 weeks and fetal heart tones present at the time of admission. If the fetal heart tones are absent, the pregnancy can be ignored, and the treatment should be focused on the mother. If fetal distress is recognized, an emergency cesarean section must be completed [2]. Emergency cesarean section due to trauma performed beyond 25 weeks is associated with 45% fetal survival [5]. Surgery is usually the treatment of choice when it comes to gunshot wounds during pregnancy [1,3,10]; however, certain patients could benefit from conservative treatment, which includes proper reanimations and clinical surveillance. This is recommended when the mother is hemodynamically stable, the bullet wound is below the fundus, the bullet is radiologically anterior to the posterior uterine walls, there is no hematuria or hematochezia, and the fetus has not survived and could be delivered later [[1], [2], [3],^7^,^9^]. Nevertheless, if a viable fetus is thought to be endangered, as it was the case with our patient, laparotomy and abdominal delivery offer the best chance for fetus survival [1,3,9].

## 4 Conclusions

Although traumas by gunshots are rare in pregnant women, they imply a notable public health burden and a complex scenario for healthcare providers. Adequate and timely treatment could benefit a viable fetus and diminish serious maternal morbidity and mortality. Management of trauma to the pregnant women requires a multidisciplinary and coordinated strategy involving trauma surgeons, emergency medicine physicians, obstetricians, and neonatologists. Training in maternal and infant resuscitative measures and surgical techniques are of vital importance for all trauma physicians.

## Conflict of interest

The authors declares that there is no conflict of interest regarding the publication of this article.

## Sources of funding

The authors have no funding to report.

## Ethical approval

The authors declare that =we obtained permission from the ethics committee in our institution.

## Consent

The authors declare that written consent was obtained from the patient before publication of this case.

## Author contribution

1) Gabriel Alejandro Molina Proaño: Conceptualization; Data curation; Formal analysis.
2) William G. Aguayo: Conceptualization.
3) Jaime M. Cevallos: Conceptualization.
4) Patricio Fernando Gálvez Salazar: Conceptualization; Data curation; Formal analysis.
5) Juan Francisco Calispa Espín: Project administration, Supervision.
6) Kevin Andres Arroyo Maldonado: Project administration, Supervision.
7) Lenin J. Guzmán: Project administration, Supervision.
8) María Mercedes Cobo Andrade: Original draft; Writing - review & editing.
9) Bernardo Miguel Gutierrez Granja: Original draft; Writing - review & editing.
10) Ronald Tanner Toapanta Morales: Roles/Writing - original draft; Writing - review & editing.
11) María Monserrate Briceño Kirby: review & editing.

## Registration of research studies

The authors declare that the patient gave his consent to publish this case, and as this is a case report not human participants were involved in a study.

## Guarantor

Gabriel A. Molina MD.

## Provenance and peer review

Not commissioned, externally peer-reviewed.

## CRediT authorship contribution statement

**Gabriel A. Molina:** Conceptualization, Data curation, Formal analysis. **William G. Aguayo:** Conceptualization. **J. Marcelo Cevallos:** Conceptualization. **Patricio F. Gálvez:** Conceptualization, Data curation, Formal analysis. **Juan F. Calispa:** Project administration, Supervision. **Kevin A. Arroyo:** Project administration, Supervision. **Lenin J. Guzmán:** Project administration, Supervision. **María M. Cobo:** Visualization, Writing - original draft, Writing - review & editing. **Bernardo M. Gutierrez:** Visualization, Writing - original draft, Writing - review & editing. **Ronald T. Toapanta:** Visualization, Writing - original draft, Writing - review & editing. **María M. Briceño:** Writing - review & editing.
